# Healthcare provider and service user perspectives on STI risk reduction interventions for young people and MSM in the UK

**DOI:** 10.1136/sextrans-2018-053903

**Published:** 2019-07-26

**Authors:** Anupama Roy, Carina King, Richard Gilson, Daniel Richardson, Fiona Burns, Alison Rodger, Laura Clark, Alec Miners, Alex Pollard, Sarika Desai, Julia Bailey, Maryam Shahmanesh, Carrie Llewellyn

**Affiliations:** 1 Department of Primary Care & Public Health, Brighton & Sussex Medical School, Brighton, UK; 2 Institute for Global Health, University College London, London, UK; 3 Department of Public Health Sciences, Karolinska Institutet, Stockholm, Sweden; 4 Sexual Health & HIV, Brighton & Sussex University NHS Trust, Brighton, UK; 5 Sexual Health & HIV Medicine, Brighton & Sussex Medical School, Brighton, UK; 6 Infection & Population Health, University College London, London, UK; 7 Health Services Research and Policy, London School of Hygiene and Tropical Medicine, London, UK; 8 Blood Safety, Hepatitis, Sexually Transmitted Infections (STI) and HIV Service, National Infection Service, Public Health England, London, UK; 9 Primary Care & Population Health, University College London, London, UK

**Keywords:** STI, young people, MSM, risk reduction, intervention

## Abstract

**Objective:**

Behavioural interventions have been shown to reduce sexual behaviours associated with increased risk of sexually transmitted infections in young people (<25 years) and men who have sex with men (MSM) internationally, but evidence from England is limited. We aimed to explore service provider and user experiences and perspectives on behavioural interventions to reduce sexual behaviour risks, and the use of automated methods to triage individuals to these services.

**Methods:**

We conducted a sequential mixed methods study with sexual health service providers and users in 2015/2016. Qualitative interviews with providers and service users (heterosexual young people and MSM) in London and Brighton allowed us to explore a range of experiences and expectations. A subsequent national web-survey of service providers measured the feasibility of delivery within existing resources and preferences for intervention attributes.

**Results:**

We conducted 35 service user (15 heterosexual young people; 20 MSM) and 26 provider interviews and had 100 web-survey responses. We found considerable heterogeneity in prevention services offered. Service users and providers were broadly supportive of tailoring interventions offered, but service users raised concerns about automated, data-driven triage, particularly around equity and fairness of service delivery. Digital technologies, including social media or apps, were appealing to providers, being less resource intensive. However, one-to-one talking interventions remained popular with both service users and providers, being familiar, trustworthy and personal. Key tensions between desirability of interventions and availability of resources to deliver them were acknowledged/recognised by providers and users.

**Conclusion:**

Overall, behavioural interventions to reduce sexual behaviour risks were viewed favourably by service providers and users, with key considerations including: privacy, personalisation and convenience. However, introducing desirable targeted interventions within heterogeneous sexual health settings will require resources to adapt interventions and research to fully understand the barriers and facilitators to use within routine services.

## Introduction

Sexually transmitted infections (STIs) continue to impose a significant burden on health and health systems in England, with cases of syphilis and gonorrhoea increasing 20% and 22% respectively in 2017.^1^ This is particularly the case in young people (defined as <25 years) and men who have sex with men (MSM).^1^ Behavioural interventions can modify behaviours such as increasing condom use and STI testing, with some showing modest impacts on STI diagnosis rates.^2–4^ The UK National Survey of Sexual Attitudes and Lifestyles (NATSAL) found that a large proportion of individuals reporting sexual behaviours associated with increased risk of STI acquisition had attended sexual health clinics (SHC).^5^ Thus, SHCs provide an opportunity to offer effective targeted behavioural interventions, such as one-to-one counselling,^6 7^ directing patients to digital interventions or by displaying informational videos in waiting areas.^8 9^ However, a systematic review from 2014 of brief behavioural interventions involving young people or MSM found only three effective trialled interventions from the UK, none of which targeted MSM, highlighting the need for intervention adaptation prior to implementation.^2^

The rational targeting of interventions to those most at need may be key to demonstrating cost-effectiveness at scale. There has been a growing recognition of the potential to harness ‘big data’ for more efficient and effective targeting of health promotion.^10^ This is particularly important in sexual health, in a period of considerable budget restrictions.^11^ Exploiting routine sexual health data^12^ could provide a mechanism to triage service users into tailored health promotion interventions.

However, there is uncertainty about which evidence-based interventions could be adapted to the English setting and how to target these. The Medical Research Council (MRC) framework for complex behavioural interventions recommends formative research and pilot studies to iteratively adapt and develop context appropriate interventions.^13^ This paper presents the first step in this process, with the aim of exploring sexual health service provider and user perspectives on opportunities and challenges to implementing targeted, evidence-based behavioural interventions, including the use of an automated triage method.

## Methods

We conducted a sequential mixed qualitative and quantitative study with healthcare providers and service users in England between July 2015 and July 2016, as part of a larger feasibility study (Santé Project; ISRCTN 16738765).^14^

### Design

Face to face semi-structured interviews with service users and telephone semi-structured interviews with healthcare providers were conducted to explore users’ and providers’ experiences and expectations of risk-reduction interventions. This was followed by a quantitative web-survey of all SHCs in England, to explore barriers and facilitators to intervention and triage implementation in clinics. A concurrent discrete choice experiment questionnaire administered to service users (this has been published elsewhere) was conducted to explore service user preferences of intervention attributes.^15^ The qualitative interviews informed the design of the quantitative tools (eg, multiple choice responses for intervention barriers and intervention attributes), to quantify opinions on preferences and feasibility of intervention delivery.

### Population

For the qualitative interviews, service users were purposively recruited from two clinics in London and Brighton, with a minimum target of 15 MSM aged 16 and above and 15 heterosexuals aged 16–25 years old. The groups were stratified by age and for young people, also by gender. Participants were approached in the SHCs by research staff and provided with study information. Participants were offered a £20 voucher for taking part, and interviews were either scheduled for the same day or another mutually convenient time.

We aimed to recruit 30 service providers across England, including service leads, health advisors, doctors and nurses. Six clinics were purposively recruited, to include particular patient populations or known experience of interventions. The remaining SHC were stratified by geography, patient mix, clinic size and services provided and selected using simple random sampling. The clinic list was derived from those submitting Genitourinary Medicine Clinic Activity Data (GUMCAD) to Public Health England (PHE) in 2014. Participants from clinics run by Brook, a sexual health charity, were offered a £20 voucher for participation.

### Qualitative interviews

Following written informed consent, service users completed a brief demographic questionnaire. Interviews were conducted in person, in a private room within the SHC. The interviews covered their experience of attending a SHC, their own risk perceptions and the triaging of services within this context and acceptability of different formats of sexual risk-reduction behavioural interventions (online supplementary file S1). Interviews specifically explored users’ view of videos in clinic waiting rooms, websites and mobile phone apps, peer group sessions and individual sessions with a healthcare provider (HCP) (including single or multiple sessions). These behavioural interventions were identified from a systematic literature review.^2^

10.1136/sextrans-2018-053903.supp1 Supplementary data

Healthcare providers were invited by email to participate in interviews. Up to three invitations were sent before classifying the individual as non-responsive and a new service randomly sampled. Following a positive response, we sent the full study information sheet and arranged a time for a telephone interview. The interviews covered services currently offered by their clinic, their opinions on triaging and targeting services using automated methods and the feasibility of delivering different behavioural interventions in their setting (online supplementary file S2).

10.1136/sextrans-2018-053903.supp2 Supplementary data

Interviews took approximately 30 min. All interviews were audio-recorded, then transcribed verbatim by an external medical transcription service and any identifying information removed. The interviewers were all female (Anupama Roy (AR), Carina King(CK) and Sarika Desai (SD)) and had received training on conducting qualitative interviews and analysis, including mock interviews with members of the Santé Patient Public Involvement group and Project Management Team, which included clinicians working in SHC.

### Web survey

For the survey, a list of SHC in England who submitted data to PHE through GUMCAD in 2014 was used. Email contacts were collated from service websites, GUMCAD contacts and a previously collated list.^16^ Clinics were invited to participate in the web-survey by email, with up to three emails sent in the first round of recruitment (November–December 2015). A second round was sent in March 2016, using individually addressed emails to service leads. A third round of recruitment was completed in June 2016 through leaflets and posters advertising the web-survey at the 2016 BASHH conference.

Prior to distribution, the survey was piloted by three clinical researchers. The web-survey was delivered using Opinio and did not include collection of any identifiable information about the respondent; providing the clinic name was optional. The first page of the web-survey included study information, and consent was implied by participation. The survey used multiple choice questions (with a space for additional free text comments) and branching logic, depending on the respondents’ responses. Answering all possible questions took approximately 12 min. Questions covered the following topics: current triage approaches; perception of automated triage; electronic patient record (EPR) system; behavioural interventions currently or previously offered; perceptions of behavioural interventions not offered.

### Analysis

Qualitative data were analysed thematically using a framework approach.^17^ Transcripts were independently reviewed and coded by CK and AR. Codes were based on predetermined questions and emergent themes were identified during the coding process and prospectively applied to subsequent transcripts. Coding was conducted separately for the service user and provider interviews. Following independent coding, the researchers discussed the interpretation for service users and providers together through a face to face meeting, triangulating common themes and exploring discrepancies. The interpretation was shared with the wider study team and then revised through discussion to reach consensus.

Survey data were described using proportions for binary and categorical data and means and medians for continuous data. Data were stratified by service type (Level-3 vs Level-2 and Level-1). Level-3 services offer complete STI testing and treatment services; Level-1 and Level-2 services offer a more limited testing and treatment service, excluding complex symptomatic cases.

### Ethics

Ethical review was conducted by Westminster National Research Ethics Committee (15/LO/0690) and the University College London Research Ethics Committee (14/0835).

## Results

We conducted 35 service user and 26 provider interviews (table 1). There were 100 web-survey responses representing 145 clinical services (response rate: 145/570, 25%), with 80% from Level-3 services (table 2), representing a higher response rate than from Level-2 and Level-1 services (31% vs 7%). Findings were combined and presented under the following headings: experiences and perceptions of triage; experience and perceptions of interventions. We did not find any consistent differences in perceptions of triage or intervention types between young people and MSM, although there was divergence in digital intervention preferences between older and younger respondents.

**Table 1:** Service provider and service user interview participants

Service user (N=35)		Service providers (N=26)	
Young people (n=15)	MSM (n=20)	Level-2 (n=8)	Level-3 (n=18)
Age group		Job title	
16–20 years=8 21–25 years=7	16–25 years=7 26–50 years=6 >=51 years=7	Nurse=5 Doctor=3	Nurse=1 Doctor=10 Health advisor=7
Ethnicity		Location	
White British=10 White other=3 Black African=1 Asian British=1	White British=11 White other=2 Black British=2 Black other=1 Chinese=1 Missing=3	London=3 Non-London=5	London=9 Non-London=9
Gender			
Male=7 Female=8			

MSM, men who have sex with men.

**Table 2:** Description of web-survey respondent services and current intervention and triage approaches reported by Level-2 and Level-3 services in England

	**Level-3 (N=80**)	**Level-2 or Level-1 (N=20**)
Service description		
Services provided		
Contraceptives	80 (100%)	19 (95%)
STI testing	79 (99%)	18 (90%)
Postal STI testing	34 (43%)	12 (60%)
Post-exposure prophylaxis	75 (94%)	3 (15%)
Drug/alcohol clinic	14 (18%)	5 (25%)
Young person’s clinic	54 (68%)	11 (55%)
MSM clinic	29 (36%)	1 (5%)
Staff available		
Health advisors	66 (86%)	6 (32%)
Counsellors	20 (25%)	2 (15%)
Psychologists	26 (33%)	4 (20%)
Drug/alcohol advisor	15 (19%)	2 (20%)
Outreach	30 (38%)	3 (15%)
Current EPR and triage*		
Clinic has an EPR system	54 (82%)	14 (88%)
EPR has ever been amended		
Never tried	3 (5%)	6 (38%)
Unsuccessful attempt	4 (6%)	0
Not sure	9 (14%)	1 (6%)
Amended	38 (58%)	7 (44%)
Risk triaging is conducted	53 (80%)	10 (63%)
Timing of triage†		
Online	7 (13%)	2 (20%)
At reception	12 (23%)	3 (30%)
During consultation	51 (96%)	10 (100%)
Key variables considered in triage		
Age	16 (30%)	3 (30%)
Gender	0-	0
Sexual orientation	23 (43%)	4 (40%)
Ethnicity	0-	0
Prior STI diagnosis	8 (15%)	1 (10%)
Number of partners	29 (55%)	8 (80%)
Condom use	12 (23%)	4 (40%)
Alcohol use	11 (21%)	2 (20%)
Drug use	18 (34%)	3 (30%)
Triage approach		
Healthcare provider	22 (42%)	3 (30%)
Proforma	3 (6%)	2 (20%)
Provider and proforma	26 (49%)	5 (50%)
Patient preference	1 (2%)	0
Algorithm	1 (2%)	0
Behavioural interventions		
Currently offered		
Leaflets	65 (81%)	15 (75%)
Videos	3 (4%)	1 (5%)
Online	8 (10%)	5 (25%)
App	2 (3%)	0
One to one‡	56 (70%)	11 (55%)
Multiple one to ones	38 (48%)	2 (10%)
Group sessions	7 (9%)	5 (25%)
No longer offered (but used to be)		
Videos	4 (5%)	1 (5%)
Online	0	1 (5%)
App	1 (1%)	0
One to one	2 (3%)	0
Multiple one to ones	3 (4%)	2 (10%)
Group sessions	5 (6%)	1 (5%)

*N=82 as not all respondents completed the survey.

†Categories are not mutually exclusive.

‡Refers to either face to face or via telephone, and these categories were not distinguished in the survey.

EPR, electronic patient record; MSM, men who have sex with men; STI, Sexually transmitted infection.

### Perceptions and experiences of triage

From the survey, 77% of respondents reported currently operating a triage system. Clinics reported a range of approaches to triaging into health promotion interventions and different clinical pathways. The most common was a combination of proforma plus provider judgement (50%), with one Level-3 clinic reporting using an algorithm (table 2). Healthcare providers gave varied examples of triage rules, for example, ‘MSM with greater than X number of partners’ (Participant 1, Doctor, Level-3). One interview respondent reported using an electronic triaging system; however, standardised systems were not the norm: ‘We have sort of GUM guidelines, departmental guidelines, but it's down to the individual doctor or nurse seeing the patient to decide whether someone should see the health adviser’ (Participant 4, Doctor, Level-3). Interestingly, no service users perceived being triaged during their clinical visit.

We presented the concept of automated data-driven triage, using patients’ routine electronic data to predict their STI risk. This was widely seen by providers as something which was already done implicitly, resulting in some respondents questioning the utility and resource implications: ‘if anything was going to be developed that had a chance of being used it would have to not increase the length of time… you know, it shouldn’t interfere with the flow of patients’ (Participant 10, Doctor, Level-3). Others thought it was acceptable: ‘I think that would work because we do triage forms which give us a little bit of a clue’ (Participant 19, Health Advisor, Level-3).

Service users presented mixed views on triage. On the positive side, the process of having a score could potentially act as an intervention: ‘it’s something that people might not like, but you, kind of have to know, it’s better to know’ (Female, 23 years, Brighton). A key factor in accepting this approach was service user trust of healthcare providers knowing best: ‘you’re a registered healthcare professional, so I trust your reasoning’ (Heterosexual male, 18 years, Brighton). This also predicated on the triage being well-explained (ie, no ‘technical jargon’). Conversely, while acknowledging awareness of being ‘high-risk’ as useful, not all would be receptive: ‘I wouldn’t consider changing my behaviour actually, I would just see it as, yes, a warning’ (MSM, 22 years, London). Similarly, some felt it would be redundant, telling them something they already ‘know’: ‘either way I'm going to get tested, so I don’t know why they tell people really’ (Female, 23 years, London). An MSM participant highlighted the potential for feeling pigeon-holed for being from a certain demographic: ‘with gay culture being so sleazy you just sort of expect to be high risk all the time’ (MSM, 23 years, London).

Many service users expressed concerns about restricting access to interventions, but were conscious of the potential need for this due to resource limitations. Barriers to the success of automated triage from the provider’s perspective included time, resources, score reliability, training requirements and issues in adapting EPR systems. In the web-survey, four services reported being unable to amend their EPR system (table 2). On the other hand, standardisation, benefits of accurate prediction and patient acceptability were highlighted as opportunities.

### Perceptions and experiences of behavioural interventions

#### Videos in waiting rooms

Mixed views were expressed by service providers and users on educational videos in SHC waiting rooms. For providers, the ease of broadcasting a message to a captive audience was the primary motivation reported in support of this intervention in the web-survey (table 3), although very few services reported currently or ever displaying videos (table 2). An important issue raised was the lack of targeting and appropriateness for clinics catering for diverse populations: *‘*we have a very heterogeneous waiting room for the walk-in clinic, you know. The challenge, I guess, would be how you target that, or do you have a number of different ones for different risk groups*’* (Participant 7, Doctor, Level-3). This was echoed by service users who felt videos could make people feel awkward in mixed waiting rooms or increase anxiety. Service users expressed the view that education was generally positive good idea, and SHCs were the correct setting for sexual health education: ‘Why not?’ Information is a good thing. It’s a sexual clinic, so that’s why people are there, to talk about sex’ (MSM, 46 years, London). However, the content of videos was contested, with some desire for ‘shock tactics’, while most respondents suggested content should focus on statistics, text information and short advertisement or campaign style clips.

**Table 3:** Desirable interventions and their perceived benefits and current barriers to implementation in Level-2 and Level-3 sexual health services in England—responses to a national web-survey

	N	Desired (n, %)	Not desired (n, %)	Barrier	Motivation
Educational videos	79	35 (44%)	14 (18%)	Lack of funding for development (37%)	Captive patient audience (37%)
Online learning materials	71	48 (68%)	7 (10%)	Lack of funding for development (61%)	Minimal staff time (33%)
Mobile ‘app’	80	49 (61%)	3 (4%)	Lack of funding for development (65%)	Potential patient uptake (47%)
One to one	20	8 (40%)	3 (15%)	Time constraints (50%)	Widely appropriate for patients (38%)
Multiple sessions of motivational interviews	45	16 (36%)	4 (9%)	Lack of funding for staff (50%)	Perceived effectiveness (50%)
Group sessions	71	16 (23%)	32 (45%)	Lack of trained staff time (38%)	Encourages peer learning (50%)

#### Online and digital interventions (including ‘apps’)

Among web-survey respondents, only 15% reported offering referrals to apps or webpages (table 3). However, service users commonly reported searching the internet for sexual health information, being convenient and easily accessible; this was echoed by providers: *‘*We have quite an IT-savvy patient group, I would say, so something like that might appeal*’* (Participant 14, Health Advisor, Level-3). However, the ‘Google effect’ was apparent among service users: ‘you can go from headache to brain tumour in 2 minutes’ (MSM, 21 years, Brighton), highlighting the need for reliable and trustworthy online resources, such as NHS branded content.

Respondents expressed divergent views on their preferred digital intervention format, that is, websites, apps or social media. For services users, concerns about apps in particular came from younger respondents, with phones being a social rather than private possession: ‘people use my phone, so they would know my business’ (Female, 20 years, Brighton). In addition, apps were considered somewhat redundant if there was a website: ‘most information I can find it online, I don’t need an app just for that […] it’s not like you need to check it every day’ (MSM, 24 years, London). Integration into a more general health app or one that could book appointments was considered convenient. This contrasts with the enthusiasm that service providers expressed: *‘*Yeah, well, they love apps. I mean we suggest apps. I’m quite an elderly nurse now but even I know to suggest an app*’* (Participant 16, Nurse, Level-2).

Use of social media for digital interventions raised concerns over anonymity, although not unanimously, with some MSM reporting they had ‘followed’ social media pages about sexual health. More specifically, apps and websites targeted at MSM such as Grindr were suggested as convenient for sexual health information. However, from the provider’s perspective the lack of perceived patient motivation was a barrier for uptake: *‘*There’s so much else to distract them on the internet, but unless it’s something they enjoy doing, the learning is not going to happen unless it’s couched in a very user-friendly, quick, vehicle’ (Participant 11, Doctor, Level 3). Moreover, as the web-survey suggests, the need for resources to develop and maintain this intervention type was a key barrier (table 3).

#### One-to-one interventions

One-to-one sessions with a clinical service provider (either in person or by phone), including motivational interviewing over multiple sessions, are offered by 67% of services, second only to providing leaflets (80%) (table 2). However, providers highlighted considerable challenges with offering one-to-one counselling, including lack of evidence and resourcing: ‘*Commissioners, I think, will not fund anything that hasn’t been shown to be effective. And so I think you’ll have to demonstrate in some way that it is effective and not just that it’s acceptable*’ (Participant 10, Doctor, Level-3).

Service providers were concerned about the amount of trained staff time needed to deliver one-to-one sessions; this was particularly pertinent to interventions involving more than one session (*‘*we’ve never had capacity to do that*’* Participant 5, Health Advisor, Level-3). Despite these concerns, many providers felt that brief sessions were effective, even if this was hard to demonstrate: *‘*Yes I know that's probably not the most cost efficient. But I think that’s probably the most effective method of risk reduction, because it is tailored to the actual patient’s needs and you have time to explore what their risk is*’* (Participant 21, Health Advisor, Level-3).

From the web-survey, of SHCs offering one-to-one sessions, half reported referring <10% of patients, but two clinics (3%) referred more than 50% of their patients to one-to-one sessions. Nearly a quarter (24%) of clinics felt they had capacity to refer more, while 41% reported being over capacity. Of clinics that reported no longer offering a particular intervention (n=20), a lack of funding and staff time was given as the reason in all but three instances, with poor patient uptake (groups), lack of impact (video) and lack of materials (video) as the other reasons. Overwhelmingly the funding for one-to-one sessions (92%) came from core service budgets, while video, app and online interventions either cost nothing (eg, existing NHS webpages) or were funded through research grants or charity initiatives.

The key benefits of one-to-one sessions, namely tailoring content to an individual patient and perceived privacy were highlighted by service users, with human interaction being key: ‘the thing about, you know, chatting to a human is, they're receptive’ (MSM, 20 years, London). Having the space and opportunity to ask questions was also seen as important, but service user expectations for sessions were linked to their trust of the healthcare providers who made referrals or delivered sessions.

#### Group interventions

On the whole, group interventions conducted within SHCs were not well received by providers or service users (eg, *‘*I think that’s a non-starter*’—*Participant 18, Health Advisor, Level-3). Concerns centred around privacy and not wanting to share personal, and potentially embarrassing, information with a group: ‘you share funny stories with your friends, and I do talk about sex quite a lot with my friends, but not about this part’ (Female, 22 years, London). This partly stemmed from a perception that the content might not be immediately relatable to them or participants would be judged by other service users. Despite these concerns, 12% (n=12) of web-survey responses reported currently offering group sessions, and six additional clinics reported previously offering this intervention.

## Discussion

We conducted a mixed-methods evaluation of the desirability and acceptability of targeted evidence-based behavioural interventions in routine SHCs in England, to inform the feasibility of testing their effectiveness in these settings. We found that current SHC provision is heterogeneous, with both ad-hoc triage and a wide variety of interventions offered, suggesting that adaptation of existing evidence-based interventions may face challenges with standardisation. While service users and providers were broadly in support of tailored interventions, there were concerns about automated data-driven triage. We found that modern technologies (eg, apps) for health promotion were appealing for several reasons, such as requiring fewer clinic resources, but there were concerns with privacy and engagement. One-to-one sessions with health advisors were popular, being both familiar to service providers and offering personalised advice and privacy to service users.

In the UK, the National Institute for Health and Care Excellence (NICE) currently recommends that one-to-one interventions for high-risk individuals are provided in SHC to support sexual risk reduction and behaviour change.^18^ Despite this, we found that while many clinics offer one-to-one sessions, this was not universal and the methods used to target these to individuals with high-risk sexual behaviours were inconsistent. The capacity to deliver one-to-one sessions varied, compromising the ability to standardise the targeted delivery of interventions. Initiatives to move sexual health service provision online^19–21^ could provide an opportunity for standardisation and links to digital resources. Using self-reported data on recent sexual behaviours, service users could be directed to different interventions, including postal self-testing, or face-to-face appointments in a SHC. Service user self-triage has been shown to be feasible and reliable in other settings.^22^

Moves towards digitalisation of sexual health services need to recognise the tensions we observed between the convenience of digital innovations vs the familiarity and perceived privacy associated with one-to-one interventions conducted in person. In this study, providers expressed more enthusiasm for digital innovation than service users, who highlighted the importance of a more holistic approach to the individual (‘personal touch’). This was echoed in a discrete choice experiment where, on average, service users had a preference for one-to-one talking interventions with clinical staff, rather than digital and group interventions.^15^ Central to this is the need for interventions to be personal, private and trustworthy, which can be achieved but many digital interventions currently do not fulfil.^4 23^ Importantly, these factors seemed to outweigh convenience which previous studies have highlighted.^24 25^ Among young people in the 2010 NATSAL survey, the internet was only reported as a source of sexual health information by 29% of men and 14% of women;^26^ however, we found all service users reported accessing the internet for sexual health information. With the drive for technology-based solutions to improve healthcare service efficiency,^27^ careful evaluation will be needed to ensure that this does not impact negatively on engagement with services, especially among higher risk individuals.

Several tensions and contradictions were apparent between the priorities of service users and providers, in an era of limited resources. Service user concerns with automated triaging revolved around being denied services, while providers were concerned with the practicalities of implementation and accuracy of a data-driven approach. There has been a shift in policy towards harnessing ‘big data’ in health promotion;^28^ however, previous research found that use of algorithms may face resistance from service providers if they are not transparent.^29^ While in principle, service users understood the issues around limited resources and providers already routinely tailor patient pathways, data-driven approaches need to be transparent and accurate to be trusted. BASHH guidelines recommend key variables for triaging patients,^30^ which correspond to those reported in the web-survey, and may therefore be a sensible basis for generating algorithms. However, whether these can be implemented with existing EPR systems and perform with the same sensitivity and specificity across clinical settings is uncertain.^31^

A key strength of this study was the theoretical basis for our approach, following the MRC recommendation for developing and evaluating complex interventions.^13^ The qualitative interviews were based on evidence from a systematic literature review, to identify brief interventions that have shown effectiveness at either increasing STI testing, reducing risky sexual behaviours or reducing STI diagnoses.^2^ Using mixed methods with both service providers and users, allowed us to triangulate and explore tensions around desirability and acceptability; however, there are limitations. First, the low response rate for the provider web-survey. We encountered several issues in sending the web-survey to all SHCs in England in the absence of any current central register of contacts. This was particularly an issue for services tendered by local authorities to private providers. Additionally, Level-3 services were more likely to respond than Level-1 and Level-2 services; therefore, the data are biased towards clinics offering more comprehensive services. Second, service users were only recruited from two clinics, both large Level-3 services located in southern England and may therefore not reflect the full diversity of service users across England. Third, Level-2 providers were under-represented in interviews, and we were unable to recruit our initial target. The interviews were all conducted by three female researchers, which may have influenced responses, particularly among MSM or heterosexual men. We conducted pilot interviews to ensure our language and interview approach was acceptable to all respondents to mitigate this potential bias.

We saw variation in services offered, but similar preferences and concerns raised by providers and service users, focusing on privacy, accessibility, convenience and personalisation to individual needs, all of which need to be balanced with resource availability. Overall, one-to-one sessions were closest to meeting these needs. However, it is important to note that the quality and content of one-to-one sessions, including fidelity to motivational interviewing approaches, needs to be rigorously evaluated to determine effectiveness at scale. There was a lack of agreement between service users and providers on the role and scope of online and digital interventions and hesitation around the role of automated triage. As sexual health service cuts continue to restrict the scope of face to face services^11^ and incorporate digital pathways, resolving this contradiction is crucial to planning service implementation and ongoing monitoring. Using the MRC framework for complex interventions, the next step is to adapt the potentially acceptable targeted one-to-one and digital interventions and pilot this with routine care settings, to assess feasibility prior to conducting a large-scale trial.

**Key messages**

- Behavioural interventions to reduce high risk sexual behaviours exist, but there is an evidence gap regarding their appropriateness for routine sexual health services in England.
- Data-driven triage, while generally acceptable, raised concerns regarding restricting access to services, accuracy of the tool and practicalities of use within electronic patient record systems.
- Behavioural interventions to reduce high risk sexual behaviours were seen favourably by both service providers and users, with key considerations including privacy, personalisation and convenience.
- There is considerable heterogeneity in behavioural interventions offered, resources available and the way service users are triaged in sexual health services in England.

## References

[1] PublicHealth England Sexually transmitted infections and screening for Chlamydia in England Health Protection Report. 12, 2017.

[2] Long L, Abraham C, Paquette R, et al Brief interventions to prevent sexually transmitted infections suitable for in-service use: a systematic review. Prev Med 2016;91:364–82 https://doi.org/ 10.1016/j.ypmed.2016.06.038 27373209

[3] Flowers P, Wu O, Lorimer K, et al The clinical effectiveness of individual behaviour change interventions to reduce risky sexual behaviour after a negative human immunodeficiency virus test in men who have sex with men: systematic and realist reviews and intervention development. Health Technol Assess 2017;21:1–164. 10.3310/hta21050 PMC5303929 28145220

[4] Bailey J, Mann S, Wayal S, et al Sexual health promotion for young people delivered via digital media: a scoping review In: Research PH, Southampton UK, eds Library NJ. 3, 2015: 1–120. 10.3310/phr03130 26583166

[5] Sonnenberg P, Clifton S, Beddows S, et al Prevalence, risk factors, and uptake of interventions for sexually transmitted infections in Britain: findings from the national surveys of sexual attitudes and lifestyles (Natsal). The Lancet 2013;382:1795–806. 10.1016/S0140-6736(13)61947-9 PMC3899025 24286785

[6] Metsch LR, Feaster DJ, Gooden L, et al Effect of risk-reduction counseling with rapid HIV testing on risk of acquiring sexually transmitted infections: the aware randomized clinical trial. JAMA 2013;310:1701–10. 10.1001/jama.2013.280034 24150466 PMC4110051

[7] Roye C, Perlmutter Silverman P, Krauss B A brief, low-cost, Theory-Based intervention to promote dual method use by black and Latina female adolescents: a randomized clinical trial. Health Educ Behav 2007;34:608–21. 10.1177/1090198105284840 16740522

[8] Mevissen FEF, Ruiter RAC, Meertens RM, et al Justify your love: testing an online STI-risk communication intervention designed to promote condom use and STI-testing. Psychol Health 2011;26:205–21. 10.1080/08870446.2011.531575 21318930

[9] Carpenter KM, Stoner SA, Mikko AN, et al Efficacy of a web-based intervention to reduce sexual risk in men who have sex with men. AIDS Behav 2010;14:549–57. 10.1007/s10461-009-9578-2 10.1007/s10461-009-9578-2 19499321 PMC3128504

[10] Montori VM Big science for patient centred care. BMJ 2017;359 10.1136/bmj.j5600 29222180

[11] White C Sexual health services on the brink. BMJ 2017;359 10.1136/bmj.j5395 29192020

[12] Public Health England Genitourinary medicine clinic activity dataset (GUMCADv2) London, UK, 2013.

[13] Craig P, Dieppe P, Macintyre S, et al Developing and evaluating complex interventions: the new medical Research Council guidance. BMJ 2008;337 10.1136/bmj.a1655 PMC2769032 18824488

[14] King C, Llewellyn C, Shahmanesh M, et al Sexual risk reduction interventions for patients attending sexual health clinics: a mixed-methods feasibility study. Health Technol Assess 2019;23:1–122. 10.3310/hta23120 PMC6452239 30916641

[15] Miners A, Llewellyn C, King C, et al Designing a brief behaviour change intervention to reduce sexually transmitted infections: a discrete choice experiment. Int J STD AIDS 2018;29:851–60. 10.1177/0956462418760425 29629651

[16] Nadarzynski T, Smith HE, Richardson D, et al Sexual healthcare professionals' views on HPV vaccination for men in the UK. Br J Cancer 2015;113:1599–601. 10.1038/bjc.2015.403 26575602 PMC4705902

[17] Gale NK, Heath G, Cameron E, et al Using the framework method for the analysis of qualitative data in multi-disciplinary health research. BMC Med Res Methodol 2013;13 10.1186/1471-2288-13-117 PMC3848812 24047204

[18] National Institute for Clinical Excellence Sexually transmitted infections and under-18 conception prevention London, UK, 2007.

[19] Cleary M, O’Sullivan J P145 London sexual health transformation programme. Sex Transm Infect 2017;93(Suppl 1):A64–A65.

[20] Gibbs J, Aicken CRH, Sutcliffe LJ, et al Mixed-methods evaluation of a novel online STI results service. Sex Transm Infect 2018;94:622–4. 10.1136/sextrans-2017-053318 29326179 PMC6288705

[21] Wilson E, Free C, Morris TP, et al Internet-accessed sexually transmitted infection (e-STI) testing and results service: a randomised, single-blind, controlled trial. PLOS Medicine 2017;14:e1002479 10.1371/journal.pmed.1002479 29281628 PMC5744909

[22] Chambers LC, Manhart LE, Katz DA, et al Evaluation of an automated express care triage model to identify clinically relevant cases in a sexually transmitted disease clinic. Sex Transm Dis 2017;44:571–6. 10.1097/OLQ.0000000000000643 28809775

[23] Gibbs J, Gkatzidou V, Tickle L, et al ‘Can you recommend any good STI apps?’ A review of content, accuracy and comprehensiveness of current mobile medical applications for STIs and related genital infections. Sex Transm Infect 2017;93:234.1–5. 10.1136/sextrans-2016-052690 PMC5520270 27884965

[24] Eaton S, Biggerstaff D, Pink J, et al Factors affecting young people's preferences for emerging technologies for chlamydia testing and treatment: a discrete choice experiment in England. The Lancet 2016;388 10.1016/S0140-6736(16)32280-2 PMC6352830 30700477

[25] Selkie EM, Benson M, Moreno M Adolescents' views regarding uses of social networking websites and text messaging for adolescent sexual health education. Am J Health Educ 2011;42:205–12. 10.1080/19325037.2011.10599189 22229150 PMC3251629

[26] Tanton C, Jones KG, Macdowall W, et al Patterns and trends in sources of information about sex among young people in Britain: evidence from three national surveys of sexual attitudes and lifestyles. BMJ Open 2015;5 10.1136/bmjopen-2015-007834 PMC4360842 25743153

[27] Nadarzynski T, Morrison L, Bayley J, et al The role of digital interventions in sexual health. Sex Transm Infect 2017;93:234.2–5. 10.1136/sextrans-2016-052926 27932599

[28] Barrett MA, Humblet O, Hiatt RA, et al Big Data and Disease Prevention: From Quantified Self to Quantified Communities. Big Data 2013;1:168–75. 10.1089/big.2013.0027 27442198

[29] Aicken CRH, Armstrong NT, Cassell JA, et al Barriers and opportunities for evidence-based health service planning: the example of developing a decision analytic model to plan services for sexually transmitted infections in the UK. BMC Health Serv Res 2012;12:202–02. 10.1186/1472-6963-12-202 22805183 PMC3519719

[30] Brook G, Bacon L, Evans C, et al 2013 UK national guideline for consultations requiring sexual history taking. clinical effectiveness group British Association for sexual health and HIV. Int J STD AIDS 2014;25:391–404. 10.1177/0956462413512807 24285601

[31] King C, Hughes G, Furegato M, et al Predicting STI diagnoses amongst MSM and young people attending sexual health clinics in England: triage algorithm development and validation using routine clinical data. EClinicalMedicine 2018;4-5:43–51. 10.1016/j.eclinm.2018.11.002 31193629 PMC6537562

