# Supplementary material for: Healthcare provider and service user perspectives on STI risk reduction interventions for young people and MSM in the UK
Source: Sex Transm Infect. 2019 Jul 26;96(1):26–32. doi: 10.1136/sextrans-2018-053903 (PMC7029238; doi:10.1136/sextrans-2018-053903)
Supplement: Supplementary data [file sextrans-2018-053903supp001.pdf]

Supplementary File 1: Service user interview topic guide

1. Introduction
   a. Introduce self
   b. Explain study and objectives
   c. £20 voucher
   d. Written consent and demographic questionnaire
   e. Confidentiality
   f. Length of interview and tape recording

2. Opening questions:
   a. What brought you to the clinic the today?
   b. During your visit, did you talk to anyone about your sexual health?

3. Risk perception:

I want to ask you about your understanding of chances of getting an STI if that's ok, as people have different understandings.

   a. How likely do you think you are of getting STI? Why do you think so?
      (prompt: partner numbers, number & type of relationships, frequency of STI screens, condoms, other preventative measures)
   b. Would you say your chance of getting HIV is different? Could you expand on that a bit more?
      (prompt: number and type of relationships, partner HIV status, adoption of seroadaptive strategies, drug use, MSM parties/venues other preventative measures e.g. frequency of HIV testing, condom use)
   c. Would you say your risk of getting HIV changes over time? (if yes) How? Why?
   d. Today you would have given some information about yourself and your recent sexual behaviour. How would you feel if that information was used to tell you that your chance of getting an STI is low or high (such as alcohol screening scores)?
   e. How would you feel if you were offered some kind of support to promote your sexual health that was based on the results of the calculation I just mentioned?

4. Behavioural interventions:

I'd like to ask a bit more about your thoughts on things which you might be offered in a clinic, to support your sexual health

   a. Have you ever been given any sexual health information or received support for your sexual health, if needed? (prompt: such as brief chat with a clinical staff, or receiving a leaflet or given condom)
   b. Was there anything (you thought was) good or bad about them?
   c. Do you think any helped you, or made you change any behaviour?
   d. I'm now going to give you some examples of health promotion things that we might develop from our study. I'd like to get your thoughts on these, good and bad:
      - A brief chat with a healthcare professional? How long? How often? Where? What format e.g. phone, person, and email? Who?
      - A video on safer sex behaviours in the waiting room?
      - Talking with other service users about sexual health and ways to promote SH?
      - Online information such as online videos or quizzes or things on social media (facebook)?

- Information on a mobile phone (app/SMS)?

e. These are just examples which we are thinking about, but are there any other services or methods to provide information that you'd like or want? What would you really like?
f. Based on the last visit to a clinic, what would have made your experience nicer?
g. Given all the things we've discussed; do you think you would you actually use any of these programmes if you were offered them?

5. Conclude:

a. Is there anything else you think would be helpful?
b. Thanks and voucher
c. Re-iterate confidentiality
