# Supplementary material for: Healthcare provider and service user perspectives on STI risk reduction interventions for young people and MSM in the UK
Source: Sex Transm Infect. 2019 Jul 26;96(1):26–32. doi: 10.1136/sextrans-2018-053903 (PMC7029238; doi:10.1136/sextrans-2018-053903)
Supplement: Supplementary data [file sextrans-2018-053903supp002.pdf]

Supplementary File 2: Service provider telephone interview topic guide

1. Clinic description/pathways of care:
   a. What clinical services do you provide?
   b. Which staff groups see patients?
   c. What is a typical patient flow/ pathway through the clinic, for a male clinic attendee and a female clinic attendee?
   d. How are the STI screen results communicated to patients?
   e. Do patients with an STI come back for a test of cure? Which patients?
   f. In your clinic, how is patient sexual risk assessed/triaged?
   g. Do you run any specialist clinics?
   h. What are your referral protocols to other services?
   i. Are there any special funding programmes/initiatives currently underway?
   j. Are there any other features of your clinical provision you feel we have not addressed?

2. Health advisors:
   a. Does your clinic have any health advisors?
   b. If YES: How many? What activities do they cover, for example partner notifications or counselling?
   c. If NO: Do you have other staff which act in the same roles as health advisors? What activities do they do?
   d. How are patients referred to see the health advisors?
   e. What other staff specialties do you have in the clinic, and how many?

3. Behavioural Interventions:
   a. Do you conduct any sexual risk reduction interventions taking place, whether formally or informally?
   b. If YES, how are patients assessed for them? Who delivers them and what do they consist of? How is this paid for or resourced? Are there any particular challenges in delivering these?
   c. If NO, what are the barriers to these being delivered in your clinic?
   d. Are there any new/additional services you provide? E.g. club drug services, social marketing of condoms, outreach services etc. How are they funded?
   e. Any thoughts on services you would like to provide/have tried and have not worked in the past?

4. Our project:
   a. Would a risk assessment tool that calculated a personal risk score using routinely collected sexual risk information be acceptable in your clinic and to your clinic staff? What are the potential challenges that you foresee in using this tool?
   b. What are your thoughts on the feasibility, including any opportunities or challenges you can see, with the following behavioural intervention formats:
      - Such as a video in the waiting room
      - Group session with patients as they wait for appointments/results?
      - Online learning material for use on smart phone or computer?
      - A 15-20 minute appointment with a health advisor? (*only if they have one*)
      - A brief series (4-6) of motivational interviews with a qualified professional? In person or by phone?
   c. Any other thoughts or comments?

Note: with lead clinicians and/or service leads we will focus on sections 1,2 and 4. With Health advisor we will focus on sections 2, 3 and 4.
